# The Effects of Philosophy for Children on Children’s Cognitive Development: A Three-Level Meta-Analysis

**DOI:** 10.3390/jintelligence13100130

**Published:** 2025-10-13

**Authors:** Caiyun Wei, Lele Chen

**Affiliations:** 1 School of Education Science, Nanjing Normal University, Nanjing 210097, China; 170601020@njnu.edu.cn; 2 Faculty of Education, Henan University, Kaifeng 475004, China

**Keywords:** cognitive development, Philosophy for Children, three-level meta-analysis, higher-order thinking, community of inquiry

## Abstract

Amid the rise of the knowledge economy, accelerated informatization, and the emergence of artificial intelligence, Philosophy for Children (P4C) has been promoted as an effective educational project to enhance children’s cognitive development, especially higher-order thinking skills. However, empirical evidence regarding its efficacy remains inconclusive. This three-level meta-analysis synthesizes 53 effect sizes derived from 33 experimental and quasi-experimental studies involving 4568 participants to assess P4C’s cognitive effects and potential moderators. The results reveal a statistically significant and moderate-to-strong overall effect (*g* = 0.59). Significant and robust effects were specifically observed for reasoning, critical thinking, and creativity. Subgroup and meta-regression analyses identified sample size as a significant moderator: smaller samples tended to report larger effect sizes. Additionally, cultural context and session length showed marginally significant moderating effects. Crucially, P4C’s cognitive impact remained consistent across grade levels, research designs, and publication years, demonstrating robustness and stability across diverse implementation conditions. These findings provide updated and nuanced evidence for the effectiveness of P4C, underscoring its cross-contextual robustness and specific value in fostering cognitive abilities. Implications for policymakers, educators, and future researchers aiming to implement or investigate P4C in varied educational settings are discussed.

## 1. Introduction

In the context of the knowledge economy, rapid informatization, and the emergence of artificial intelligence, cultivating students’ cognitive competencies, especially higher-order thinking—such as critical thinking, creativity, reasoning skills, problem-solving skills, and meta-cognitive abilities—has become a central goal and shared consensus of global education reform (OECD 2010; Partnership for 21st Century Skills 2009; Gordon et al. 2009; Voogt and Roblin 2012; Reed 2020). These competencies are fundamental to individual academic success and lifelong learning, and are also regarded as essential for building a well-functioning society (Zhang 2016).

Within this context, Philosophy for Children (P4C)—an educational program and pedagogy dedicated to fostering children’s thinking skills and cognitive abilities (Lipman 2003; Yan et al. 2018) has garnered increasing global attention (Cam 2017; Gao 2018; De Cesaris 2018; Santi 2019; Michalik 2023). Rather than transmitting philosophical knowledge, P4C creates a community of inquiry where children engage in open, collaborative dialogue around a shared philosophical question, facilitated by the teacher. Aiming to form reasonable, meaningful judgments, they challenge and build on one another’s ideas, offer personal stories, attend to emotions, imagine new possibilities, and self-correct (Gregory 2021). Through Vygotsky’s (1978) concept of the “intrapsychical reproduction of the interpsychical”, children internalize the inquiry conducted in the community into the realm of their personal thinking (Lipman 2003; Kennedy and Kennedy 2011). P4C thus cultivates children’s cognitive abilities, including reasoning skills, critical thinking, creativity, meta-cognitive abilities, problem-posing and problem-solving capacities, among others.

Given its theoretical promise, numerous empirical studies have investigated P4C’s effects on cognitive outcomes. However, findings remain inconsistent, reflecting heterogeneity in design, participants, dosage, and measures. While many studies suggested that P4C enhance cognitive development (e.g., Iorio et al. 1984; Rahdar et al. 2018; Zulkifli and Hashim 2020), others reported inconclusive or non-significant results (e.g., Higa 1980; Meyer 1989; Ventista 2019; Lord et al. 2021). The inconsistency highlights the need for meta-analysis—a method that quantitatively synthesizes results from studies (Luo 2013)—to provide systematic evaluation of P4C’s cognitive effects and their moderators.

Two prior meta-analyses (García-Moriyón et al. 2005; Yan et al. 2018) provided preliminary support for its positive impact. However, they included relatively few studies (*n* = 18 and 10, respectively) and relied on conventional meta-analytic techniques, which treat all effect sizes as independent. Such an approach may ignore the statistical dependence among multiple effect sizes derived from the same sample, potentially biasing variance estimates and significance tests. Moreover, both reviews predate the recent surge of empirical research. Addressing these gaps, the present study conducts a three-level meta-analysis, which explicitly models within- and between-study variance while incorporating the expanded body of available evidence, thereby providing a more rigorous and comprehensive assessment of P4C’s cognitive value.

Accordingly, this study aims to offer a comprehensive and methodologically robust assessment of P4C’s cognitive impact through three-level meta-analysis. Specifically, we sought to address the following research questions: (1) Does P4C enhance children’s cognitive development? (2) What methodological and substantive factors moderate its efficacy?

## 2. Literature Review and Hypotheses Development

### 2.1. Philosophy for Children (P4C)

P4C is an educational project initiated by American philosopher Matthew Lipman in 1969, with the aim of “teaching children to think for themselves and make informed choices” (Trickey and Topping 2004). In 1974, Lipman and Ann Sharp established the Institute for the Advancement of Philosophy for Children (IAPC) to advance P4C.

Globally, terms like “Philosophy with Children” and “Philosophy in Schools” are used interchangeably with P4C, referring to initiatives that involve youth in philosophical discussions (Gregory 2013). Today, P4C is recognized both as a standalone curriculum and as a pedagogy integrated into other subjects (Kennedy and Kennedy 2011; Lewis and Chandley 2012).

The typical Lipman-Sharp P4C approach involves six steps (Lipman 2003; Oyler 2017): (1) stimulus presentation: such as a picture book, story, or video, often one of the IAPC-developed philosophical novels; (2) question generation: children raise and democratically select questions for inquiry; (3) facilitated dialogue: conducting inquiry dialogues facilitated by the teacher; (4) deepening inquiry: through structured exercises and discussion plans; (5) meta-cognitive reflection: participants evaluate their own and the community’s cognitive, emotional, and moral performance; (6) further response: extending inquiry through art, action projects, etc.

### 2.2. The Cognitive Value of P4C

Cognitive abilities refer to mental processes involved in the acquisition of knowledge, manipulation of information, and reasoning, as opposed to other types of skills such as motor or social skills. Cognitive abilities encompass a wide spectrum of mental processes, ranging from basic functions such as perception, attention, and memory, to higher-order abilities such as reasoning, critical thinking, creativity, problem-solving, decision-making, and meta-cognition (Carroll 1993; Kiely 2014; Gu and Hu 2018).

P4C aims to develop children’s critical, creative, and caring thinking (Lipman 2003), as well as their reasoning, moral understanding, and meaning-making capacities (Lipman et al. 1980). Over the past half-century, scholars worldwide have empirically tested P4C’s efficacy on reasoning (Säre et al. 2016), critical thinking (Cooke 2015), creativity (Kanani Harandi et al. 2021), problem-posing (Zulkifli and Hashim 2019), problem-solving ability (Işıklar and Öztürk 2022), as well as domain-specific academic competencies such as reading comprehension (Imani et al. 2016), listening comprehension (Boyraz and Ayday 2024), and math learning (Sabbagh Hasanzadeh 2024). Thus, P4C targets multiple dimensions of cognitive development.

Previous meta-analyses also confirmed P4C’s effectiveness in cognitive skills. García-Moriyón et al. (2005) synthesized 18 studies, reporting a medium effect size on reasoning. Yan et al. (2018) reviewed 10 studies, finding an overall moderate positive effect on cognition. These findings suggest that P4C exerts a robust and significant influence on children’s cognitive abilities. Accordingly, we hypothesize the following:

**H1.**  *P4C will significantly and positively affect children’s cognitive abilities.*

### 2.3. Possible Moderators of P4C’s Cognitive Effects

To comprehensively understand the effectiveness of P4C intervention, we examine potential moderators from two complementary perspectives: (1) the nature of the outcome (i.e., which specific cognitive abilities are most affected), and (2) the nature of the intervention (i.e., how variations in its delivery influence its efficacy). While these two types of moderators pertain to different parts of the causal chain (output vs. input), they are both essential for mapping the boundary conditions of P4C’s effects.

#### 2.3.1. Cognitive Ability Type

While P4C aims to cultivate a range of cognitive skills, it remains unclear whether its effects differ across cognitive domains. Systematic reviews emphasize P4C’s particular strength in promoting higher-order thinking skills—such as reasoning, critical thinking, creativity, and question-posing (Ab Wahab et al. 2022). Many studies have reported significant gains in reasoning (e.g., Lipman 1976), critical thinking (e.g., Cooke 2015), and creativity (e.g., Pourtaghi et al. 2014). However, effects on domain-specific academic skills—such as reading or math achievement—are mixed. For instance, Imani et al. (2016) and Boyraz and Ayday (2024) found improvements in reading and listening comprehension, whereas Higa (1980) and Lord et al. (2021) reported no significant academic gains. Meta-analytic evidence further supports this variability across cognition domains. Yan et al. (2018) found that P4C produced a large effect on reasoning (*d* = 1.06), moderate effects on general cognition (*d* = 0.40), and smaller effects on reading comprehension (*d* = 0.28), with significant between-group differences (Q = 15.44, *p* < 0.001). Thus, the type of cognitive ability may moderate P4C’s effects. Therefore, we propose:

**H2.**  *The type of cognitive ability will significantly moderate the cognitive effects of P4C.*

#### 2.3.2. Instructional Dosage

In the context of P4C, the intensity and duration of programs vary considerably, raising the question of whether these differences influence cognitive outcomes. Ventista (2018) notes that most P4C studies last less than one school year. Some studies suggest that short-term P4C interventions can generate cognitive gains (Lipman 1976; Pourtaghi et al. 2014; Cooke 2015; Imani et al. 2016; Işıklar and Öztürk 2022), confirmed by Yan et al. (2018), who found 5–20 h programs produce significant positive effects on students’ cognitive outcomes. However, Fair et al. (2015b) found that a 22–26-week intervention could lead to significant cognitive gains, whereas a 4–10-week program did not, recommending implementing one 60-min P4C session per week across a single semester as an effective instructional dosage. These findings suggest that dosage-related variables (session length, frequency, and overall duration) may moderate the effect of P4C. Thus, we hypothesize the following:

**H3.1.**  *The length of each P4C session will significantly moderate the cognitive effects of P4C.*

**H3.2.**  *The frequency of P4C sessions will significantly moderate the cognitive effects of P4C.*

**H3.3.**  *The overall duration of the P4C intervention will significantly moderate the cognitive effects of P4C.*

#### 2.3.3. Grade Level

Whether children at different developmental stages could engage in and benefit equally from P4C is an important theoretical and empirical debate. Piaget (1933), Kitchener (1990), and White (1992) have argued that children below the formal operational stage lack abstract thinking skills required for philosophical inquiry. While contemporary developmental psychologists, such as Alison Gopnik, have argued that young children possess more advanced cognitive capacities than Piaget originally proposed, children are endowed with abilities to form theories, test hypotheses, and reason about causality and counterfactuals from a very early age (Gopnik 2009; Gopnik and Wellman 2012), they are “philosophical babies” (Gopnik 2009) and “scientists in the crib” (Gopnik et al. 1999). P4C scholars have also provided theoretical and practical evidence that even young children can do philosophy (Matthews 1980; Lipman 1990; Murris 1999). Empirical studies generally support this opinion. For example, Demirtaş et al. (2018) found that P4C enhanced preschoolers’ ability to generate questions and give elaborated responses; Gardner (1998) observed moral reasoning gains in a K-5 longitudinal study. Meta-analytic evidence indicates consistent significant effects across grades 2–5 and 6–10 (Yan et al. 2018). Nevertheless, in order to rigorously address theoretical concerns about children’s cognitive readiness raised in the literature, it remains important to examine whether grade level systematically moderates P4C’s cognitive effects. To this end, we hypothesize:

**H4.**  *Grade Level will significantly moderate the cognitive effects of P4C.*

#### 2.3.4. Cultural Context

P4C originated in the USA and is now practiced in over 60 countries (Gregory 2021). Despite its global spread, P4C’s cultural adaptability remains an open question. Grounded in western philosophical traditions and emphasizing inquiry-based, child-centered pedagogy, the program may not fully align with all educational systems in different contexts. Yan et al. (2018) found significantly (Q = 5.16, *p* < 0.05) larger effects in non-Western (*d* = 0.69) than in Western contexts (*d* = 0.39). Cultural context is commonly examined as a moderator in meta-analyses, with the Western vs. non-Western distinction being one of the common categorizations in cross-cultural research (Triandis and Gelfand 1998; Watkins 2014; Liu and Baumeister 2016). Accordingly, culture warrants investigation as a moderator, so we hypothesize:

**H5.**  *Cultural context (Western vs. non-Western) will significantly moderate the cognitive effects of P4C.*

#### 2.3.5. Research Design

Research design has been identified as a potential moderator. García-Moriyón et al. (2005) categorized studies into three types: independent groups pretest–posttest, independent groups post-test, and single group pretest–posttest, and found that research design had a significant moderating effect. In contrast, Yan et al. (2018) classified the included studies as randomized controlled trials (RCTs) or quasi-experimental and reported no significant differences. Given these mixed findings, the moderating role of research design remains worthy of further examination. Thus, we propose:

**H6.**  *The type of research design will significantly moderate the cognitive effects of P4C.*

#### 2.3.6. Publication Year

Publication year, as a temporal variable, has been widely considered a potential moderator in meta-analyses (Nakagawa et al. 2022). Time-lag bias—where studies with larger or statistically significant effects are published more quickly than smaller or non-significant ones—may lead to a decline in reported effect sizes over time (i.e., a decline effect) (Nakagawa et al. 2022; Koricheva and Kulinskaya 2019). García-Moriyón et al. (2005) found that more recent P4C studies tended to report smaller effect sizes, and explain that this might be due to the adoption of more rigorous research designs. Given these trends, this study investigates publication year as a moderator and proposes:

**H7.**  *Publication year will significantly moderate the cognitive effects of P4C.*

#### 2.3.7. Sample Size

Sample size is a critical methodological moderator in meta-analytic research. Smaller studies are frequently associated with larger effect sizes, a phenomenon termed the small-study effect. This may stem from publication bias (i.e., the preferential publication of statistically significant results) and greater random error in small samples (Luo et al. 2020). Small-scale trials may also benefit from more favorable implementation conditions such as more intensive teacher training and higher delivery consistency, potentially leading to larger observed effects (Yan et al. 2018). Examining sample size as a moderator is therefore essential for accurately interpreting heterogeneity and enhancing the robustness of the findings. Accordingly, we propose:

**H8.**  *Sample size will significantly moderate the cognitive effects of P4C.*

## 3. Method

This study followed the Preferred Reporting Items for Systematic Review and Meta-Analysis (PRISMA) guidelines (Page et al. 2021) and was preregistered on the Open Science Framework (OSF) (registration number: 10.17605/OSF.IO/EUAPX). Coded data and scripts are openly accessible on the OSF platform.

### 3.1. Search Procedures

The final comprehensive search was conducted on 13 March 2025 using Google Scholar, Web of Science (WOS), Scopus, and ProQuest. The searching keywords were (“philosophy for children”) OR (“P4C”) OR (“philosophy with children”) OR (“P4wc”) OR (“philosophy in schools”) OR (“community of inquiry” AND “child*”) OR (“community of enquiry” AND “child*”) OR (“community of philosophical inquiry”) OR (“community of philosophical enquiry”) OR (“philosophical inquiry” AND “child*”) OR (“philosophical enquiry” AND “child*”). To minimize publication bias, both peer-reviewed journal articles and grey literature (conference papers and dissertations) were considered (Hopewell et al. 2005). All retrieved records were managed using Zotero. Duplicate records and non-English publications were removed prior to screening.

### 3.2. Eligibility Criteria and Study Selection

Potential studies were evaluated against the eligibility criteria to determine their suitability for the meta-analysis. The systematic review methodology involved establishing inclusion criteria based on the research topic, internationally recognized PICOS principles are typically adopted (Moher et al. 2009). Following the PICOS (Population, Intervention, Comparison, Outcomes, and Study design) principles, the inclusion criteria were: (1) the participants were children under 18 years old, consistent with *the United Nations Convention on the Rights of the Child* (United Nations General Assembly 1989); (2) the intervention group received the P4C curriculum or any course employing the pedagogy of P4C; (3) the control group received regular instruction without P4C components; (4) the eligible studies reported measurable outcomes regarding P4C’s impact on children’s cognitive performance, with sufficient statistics—such as sample sizes (N), means (M), standard deviations (SD), *t*-values or *p*-values—for effect size calculation; (5) studies adopted experimental or quasi-experimental designs.

Two researchers independently screened the literature through a four-stage process: (1) titles, abstracts, and keywords were reviewed to exclude records clearly ineligible based on PICOS criteria; (2) screening results were cross-verified, resolving discrepancies through discussion; (3) full texts were retrieved, and articles without accessible full texts were excluded; (4) eligible full texts underwent comprehensive review against inclusion criteria, followed by a second verification round. This dual-reviewer approach with consensus-building ensured rigor and minimized bias.

**Figure 1:**
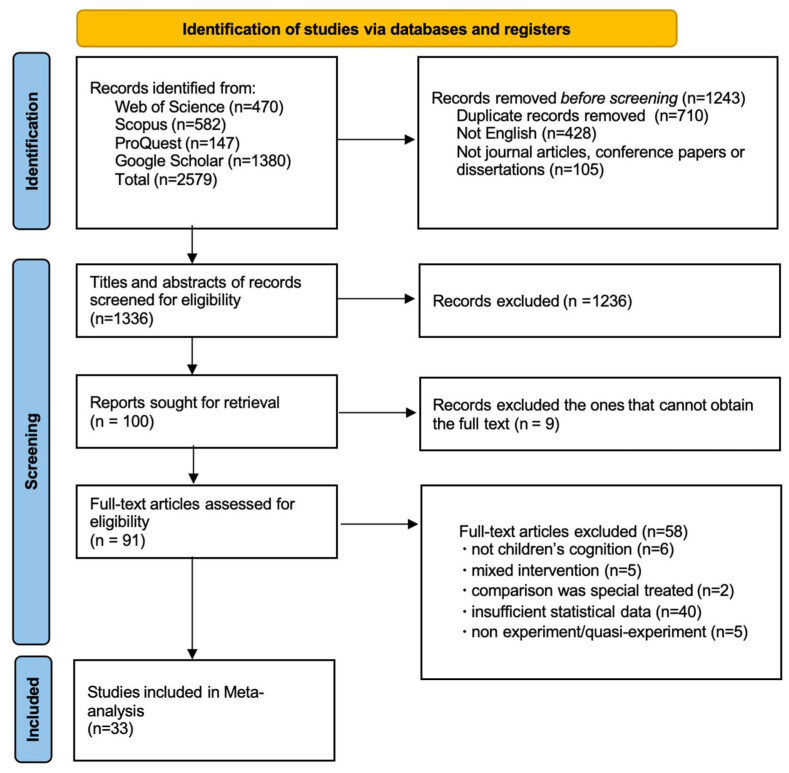
PRISMA Flow Diagram of Literature Screening.

The PRISMA flowchart of the literature screening is shown in Figure 1. A total of 2579 articles were identified. After removing 710 duplicate records, 428 non-English publications, and 105 documents that were not journal articles, conference papers, or dissertations, 1336 records remained. Titles and abstracts screening excluded 1236 records; nine studies were removed due to unavailable full texts. After full-text review based on inclusion and exclusion criteria, 58 articles were further excluded. Ultimately, 33 studies were retained, providing 53 effect sizes from 4568 participants, spanning the period from 1979 to 2023.

### 3.3. Data Extraction, Feature Coding and Quality Assessment

To ensure rigorous and reliable data extraction, two researchers independently coded the included studies. When a single study reported multiple effect sizes, each was coded separately. Extracted information included: (1) basic study information, such as first author, publication year and relevant statistical data (e.g., N, M, SD, *t*-values or *p*-values); (2) potential moderator variables, including outcome indicators (for studies reporting multiple cognitive outcomes, all relevant measures were systematically recorded), instructional dosage (session length, frequency, and total duration), grade level, country, and research design. Missing study characteristics were coded as “Not Reported” when the relevant information was unavailable in the original publications.

Coding criteria were based on both theoretical and practical considerations. Following Piaget (1933) and subsequent debates (Kitchener 1990; White 1992) regarding children’s capacity for philosophical inquiry prior to the formal operational stage (typically younger than 11–12 years, or below Grade 6), and consistent with Yan et al. (2018), grade levels were coded as ≤Grade 5 vs. ≥Grade 6.

Cultural context was coded as Western vs. Non-Western at the country level. Following prior meta-analyses and cross-cultural studies (Yan et al. 2018; Triandis and Gelfand 1998; Watkins 2014; Liu and Baumeister 2016), we classified studies as Western (USA, UK, Australia, Iceland) or non-Western (China, South Korea, Malaysia, Iran, Turkey). Western contexts are generally higher in individualism and lower in power distance, whereas non-Western contexts tend to be more collectivist and hierarchical (Hofstede 1984; Hofstede et al. 2010; Li 2012). We acknowledge that this distinction is a heuristic simplification, but it provides a pragmatic framework widely used in cross-cultural research.

Session length followed the widely adopted guideline that a standard class period lasts 40–50 min (National Health Commission of the People’s Republic of China 2012), and categorized as ≤50 min vs. >50 min. Considering a typical school semester spans approximately 20 weeks, the total duration of intervention was categorized into two groups: ≤20 weeks vs. >20 weeks. Research design was coded as randomized controlled trial (RCTs), non-randomized controlled trials (Non-RCT), or one-group pretest–posttest design.

Outcome domains were classified according to the definitions provided by the original authors and the characteristics of the instruments employed. For example, reasoning skills were frequently assessed using the New Jersey Test of Reasoning Skills (NJTRS) (Lam 2012; Marashi 2008; Pálsson 1995); critical thinking was often measured with standardized instruments such as the Cornell Critical Thinking Test (CCTT) or the California Critical Thinking Skills Test (CCTST) (Erfani and Rezaei 2016; Acar and Arslan 2023); creativity was evaluated using the Torrance Test of Creativity, as well as the “What Could You Use It For?” and “What Could It Be?” tasks (Pourtaghi et al. 2014; Cinquino 1981). General cognitive abilities were assessed with the Cognitive Abilities Test (CAT) (Fair et al. 2015a, 2015b; Topping and Trickey 2007a, 2007b), while reading comprehension was measured using a variety of instruments, including the General English Proficiency Test (Tian and Liao 2016), the Test of Reading Comprehension (TORCH) (Youssef et al. 2016), TOEFL (Othman and Hashim 2006), the reading comprehension subtest of the Comprehensive Test of Basic Skills (CTBS) (Yeazell 1982), and researcher-designed Reading Comprehension Tests (Tok and Mazl 2015).

The researchers subsequently cross-validated their coding, resolving discrepancies through discussion. Independent double coding by two researchers ensured consistent and reliable data extraction. The resulting inter-coder reliability coefficient (*Kappa* = 0.93) indicated an excellent level of agreement.

The methodological quality of the included studies was evaluated using the *Quality Assessment Tool for Observational Cohort and Cross-Sectional Studies* provided by the National Institutes of Health (NIH 2021). This tool consists of 14 items, each with five possible responses: Yes, No, Cannot Determine (CD), Not Reported (NR), and Not Applicable (NA). Scoring was performed by assigning 1 point for “Yes” and 0 points for all other responses. Studies were then classified as “good” (total score >7), “fair” (5–7), or “poor” (<5) (Zhu et al. 2024). Two authors assessed quality independently, and the inter-rater agreement was *Kappa* = 0.78.

### 3.4. Data Analysis and Synthesis

#### 3.4.1. Model Selection

Meta-analysis can be conducted with fixed-effects or random-effects models. The fixed-effects model assumes that all studies estimate a single true effect size, with variation across studies attributed solely to sampling error. However, given that the included studies differed in participant populations, cultural contexts, intervention formats, and outcome measures, it was more appropriate to adopt a random-effects model, which allows the true effect size to vary across studies (Borenstein et al. 2009). Additionally, further heterogeneity tests will be conducted to validate model choice during the subsequent analysis of the meta-analytic data.

Moreover, several primary studies included in this meta-analysis reported multiple effect sizes from the same sample, creating statistical dependence. Traditional meta-analytic methods assume independence among effect sizes and typically extract only one effect size per study (Assink and Wibbelink 2016). This approach ignores the inherent dependence, which may lead to an overestimation of the overall effect size (Lipsey and Wilson 2001). In contrast, a three-level meta-analytic model accounts for the dependency of effect sizes within the same study by partitioning the variance into three levels. Level 1 represents sampling error arising from the selection of participants within individual studies; Level 2 reflects variance among multiple effect sizes within the same study, indicating within-study heterogeneity; Level 3 captures variance among effect sizes from different studies, indicating between-study heterogeneity (Cheung 2014). Compared with traditional meta-analytic techniques, the three-level model addresses the issue of dependent effect sizes while retaining more information and increasing statistical power (Assink and Wibbelink 2016). For these reasons, the present study employed a three-level random effects model to examine the main effects, heterogeneity, moderator effects, and publication bias.

#### 3.4.2. Effect Size Calculation

Given that some included studies had relatively small sample sizes, Hedges’ *g* with 95% confidence intervals (CIs) was chosen as the measure of effect size. Compared with Cohen’s *d*, *g* includes a correction factor that reduces bias in small samples (Hedges 1981), while they yield nearly identical results in large-sample studies, making it more accurate under such conditions (Zheng et al. 2011). The interpretation of effect sizes followed Cohen’s conventional benchmarks: effect sizes less than 0.2 were considered negligible, those between 0.2 and 0.5 indicated a small effect, those between 0.5 and 0.8 indicated a medium-to-large effect, and values above 0.8 indicated a large effect (Cohen 2013).

#### 3.4.3. Heterogeneity Analysis and Moderator Analysis

The present study used one-tailed log-likelihood ratio tests to examine the variance at Level 2 and Level 3, determining whether these components are significant. If significant, moderator analyses were conducted to explore potential sources of heterogeneity (Gao et al. 2024). Moderator variables were incorporated as covariates in the three-level meta-analytic model to estimate the magnitude of their moderating effects (Gao et al. 2024). The moderator variables considered included (1) categorical moderators: cognitive ability type, dosage variables (duration, frequency, and weeks), grade level, cultural context, stimulus type, and research design and (2) continuous moderators: publication year and sample size.

#### 3.4.4. Publication Bias Assessment

Publication bias refers to the phenomenon that studies reporting statistically significant findings are more likely to be published (Franco et al. 2014). In this study, publication bias is assessed qualitatively and quantitatively using funnel plots, Rosenthal’s Fail-Safe N test, and the Trim-and-Fill method. For qualitative assessment, a symmetric funnel plot indicates low risk of publication bias (Sterne and Harbord 2004). Fail-Safe N is calculated using Rosenthal’s method, with values exceeding 5 × *k* + 10 (*k* = the number of effect sizes) suggesting no obvious publication bias, whereas smaller values indicate potential bias (Rosenthal 1979). Next, the Trim-and-Fill analysis for multilevel models is used to estimate the missing studies that could make the funnel plot symmetrical (Duval and Tweedie 2000; Fernández-Castilla et al. 2021). If R_0_^+^ > 3 and L_0_^+^ > 2 in the Trim-and-Fill analysis, publication bias existed (Fernández-Castilla et al. 2021).

#### 3.4.5. Statistical Procedures

All analyses are performed in R 4.2.0 using the metafor package (Viechtbauer 2010). R code is based on the procedures designed by Assink and Wibbelink (2016) and Rodgers and Pustejovsky (2021). Model parameters estimated using restricted maximum likelihood (REML) (Viechtbauer 2010), and statistical significance is determined at a two-tailed *p* < 0.05 level.

## 4. Results

### 4.1. Study Characteristics

The three-level meta-analysis of P4C’s cognitive effects included 33 studies with 53 effect sizes, encompassing 4568 participants from 1979 to 2023. Among the included studies, two were derived from doctoral dissertations, while the remainder were peer-reviewed journal articles. The basic characteristics of the included studies are presented in Table 1. A more detailed coding table listing the specific measurement tools used in each study has been made available on the OSF preregistration platform (https://doi.org/10.17605/OSF.IO/EUAPX, accessed on 1 October 2025). The quality assessment results indicated that the included studies were rated as either “good” (n = 31) or “fair” (n = 2).

**Table 1:** Basic information of the included studies.

ID	Year	Study (First Author)	Sample Size	Grade	Country	Dosage	Design	Outcome Indicators
						Duration/Frequency/Weeks		
1	2023	Acar (Acar and Arslan 2023)	23	S	Turkey	40 min/2 times/10 weeks	NR	CT, SK
2	2023	Şişman (Şişman et al. 2023)	34	S	Turkey	80 min/1 time/8 weeks	NR	MC
3	2022	Akbayir (Akbayir and Tedikçi 2022)	38	S	Turkey	120 min/1 time/8 weeks	NR	MA
4	2022	Işıklar (Işıklar and Öztürk 2022)	40	L	Turkey	40 min/2 times/10 weeks	NR	CT, PS
5	2021	Wu (Wu 2021)	173	S	China	40 min/2 times/4 weeks	R	CT
6	2020	Zulkifli (Zulkifli and Hashim 2020)	61	S	Malaysia	—/—/11 sessions	NR	CT
7	2020	Khanmohammadi (Khanmohammadi et al. 2020)	44	S	Iran	—/1 time/12 weeks	NR	LO, PM
8	2020	Mehnehj (Mehnehj et al. 2020)	50	S	Iran	60 min/2 time/6 weeks	NR	RS
9	2019	Ventista (Ventista 2019)	738	L	UK	30 min/1 time/40 weeks	NR	CT, C
10	2017	Abbasi (Abbasi et al. 2017)	50	L	Iran	—/—/12 weeks	NR	CT
11	2016	Tian (Tian and Liao 2016)	62	S	China	100 min/1 time/10 weeks	NR	RC
12	2016	Youssef (Youssef et al. 2016)	246	S	Australia	—/—/24 weeks	NR	RC
13	2016	Erfani (Erfani and Rezaei 2016)	40	S	Iran	120 min/1 time/12 weeks	NR	CT
14	2015	Fair (a) (Fair et al. 2015b)	275	S	USA	60 min/1 time/22–26, 4–10 weeks	R	CA
15	2015	Fair (b) (Fair et al. 2015a)	183	S	USA	60 min/1 time/22–26 weeks	R	CA
16	2015	Tok (Tok and Mazl 2015)	74	L	Turkey	120 min/2 time/10 weeks	NR	RC, LC
17	2014	Pourtaghi (Pourtaghi et al. 2014)	32	S	Iran	75 min/12 weeks	NR	C
18	2012	Lam (Lam 2012)	28	S	China	90 min/2 times/16 weeks	R	RS
19	2009	Marashi (Marashi 2008)	60	S	Iran	70 min/11 sessions	NR	RS
20	2007	Topping (a) (Topping and Trickey 2007a)	115	L	UK	60 min/1 time/58 weeks	NR	CA
21	2007	Topping (b) (Topping and Trickey 2007b)	177	L	UK	60 min/1 time/58 weeks	NR	CA
22	2006	Othman (Othman and Hashim 2006)	45	S	Malaysia	—/—/16 weeks	NR	RS, RC
23	2000	Jo (Jo 2000)	54	L	South Korea	30 min/4 times/24 weeks	NR	CM
24	1998	Sprod (Sprod 1998)	54	S	UK	70 min/1 time/40 weeks	NR	RS
25	1995	Pálsson (Pálsson 1995)	126	L	Iceland	80 min/1 time/24 weeks	NR	RS
26	1993	Chamberlain (Chamberlain 1993)	160	L	USA	60 min/5 times/12 weeks	NR	RS, HC
27	1989	Slade (Slade 1989)	50	S	Australia	120 min/12 sessions	NR	RS
28	1986	Jenkis (Jenkins 1986)	60	S	UK	45 min/about 20 weeks	NR	RS
29	1985	Martin (Martin and Weinstein 1985)	964	L, S	USA	—/—/about 32 weeks	PP	CT
30	1984	Iorio (Iorio et al. 1984)	336	L	USA	45 min/2 times/40 weeks	PP	CT
31	1982	Yeazell (Yeazell 1982)	100	L	USA	45 min/1 time/32 weeks	NR	RC
32	1981	Cinquino (Cinquino 1981)	47	L	USA	120 min/1 time/28 weeks	PP	RS, C
33	1979	Cummings (Cummings 1979)	29	L	USA	40 min/2 times/7.5weeks	NR	LT

Notes: —, not reported. Grade: L, Lower grade (≤Grade 5); S, Senior grade (≥Grade 6). Stimulus: I, IAPC-developed stimulus; N-I, Non-IAPC developed stimulus. Research Design: R, Randomized controlled trial; NR, Non-randomized controlled trial; PP, One-group pretest–posttest design. Outcome Indicators: C, Creativity; CA, Cognitive Abilities; CT, Critical Thinking; CM, Constructing Meaning; HC, Higher Cognitive Processes; L, Logic; LC, Listening Comprehension; LO, Learning Outcome; MA, Math Achievement; MC, Meta-Cognition; PM, Philosophical Mentality; PS, Problem Solving skills; RC, Reading Comprehension; RS, Reasoning Skills; SK, Speaking Skills.


**ID**

**Study**

**Sample Size**

**Grade**

**Country**

**Dosage**

**Design**

**Outcome**

**Indicators**

**Authors-Year**

**Duration/Frequency/Weeks**
1(Acar and Arslan 2023)23STurkey40 min/2 times/10 weeksNRCT, SK2(Şişman et al. 2023)34STurkey80 min/1 time/8 weeksNRMC3(Akbayir and Tedikçi 2022)38STurkey120 min/1 time/8 weeksNRMA4(Işıklar and Öztürk 2022)40LTurkey40 min/2 times/10 weeksNRCT, PS5(Wu 2021)173SChina40 min/2 times/4 weeksRCT6(Zulkifli and Hashim 2020)61SMalaysia—/—/11 sessionsNRCT7(Khanmohammadi et al. 2020)44SIran—/1 time/12 weeksNRLO, PM8(Mehnehj et al. 2020)50SIran60 min/2 time/6 weeksNRRS9(Ventista 2019)738LUK30 min/1 time/40 weeksNRCT, C10(Abbasi et al. 2017)50LIran—/—/12 weeksNRCT11(Tian and Liao 2016)62SChina100 min/1 time/10 weeksNRRC12(Youssef et al. 2016)246SAustralia—/—/24 weeksNRRC13(Erfani and Rezaei 2016)40SIran120 min/1 time/12 weeksNRCT14(Fair et al. 2015b)275SUSA60 min/1 time/22–26, 4–10 weeksRCA15(Fair et al. 2015a)183SUSA60 min/1 time/22–26 weeksRCA16(Tok and Mazl 2015)74LTurkey120 min/2 time/10 weeksNRRC, LC17(Pourtaghi et al. 2014)32SIran75 min/12 weeksNRC18(Lam 2012)28SChina90 min/2 times/16 weeksRRS19(Marashi 2008)60SIran70 min/11 sessionsNRRS20(Topping and Trickey 2007a)115LUK60 min/1 time/58 weeksNRCA21(Topping and Trickey 2007b)177LUK60 min/1 time/58 weeksNRCA22(Othman and Hashim 2006)45SMalaysia—/—/16 weeksNRRS, RC23(Jo 2000)54LSouth Korea30 min/4 times/24 weeksNRCM24(Sprod 1998)54SUK70 min/1 time/40 weeksNRRS25(Pálsson 1995)126LIceland80 min/1 time/24 weeksNRRS26(Chamberlain 1993)160LUSA60 min/5 times/12 weeksNRRS, HC27(Slade 1989)50SAustralia120 min/12 sessionsNRRS28(Jenkins 1986)60SUK45 min/about 20 weeksNRRS29(Martin and Weinstein 1985)964L, SUSA—/—/about 32 weeksPPCT30(Iorio et al. 1984)336LUSA45 min/2 times/40 weeksPPCT31(Yeazell 1982)100LUSA45 min/1 time/32 weeksNRRC32(Cinquino 1981)47LUSA120 min/1 time/28 weeksPPRS, C33(Cummings 1979)29LUSA40 min/2 times/7.5weeksNRLT

### 4.2. Assessment of Publication Bias

**Figure 2:**
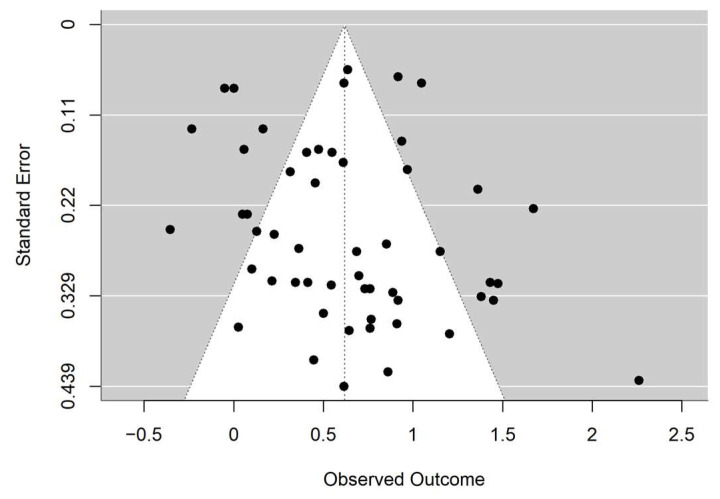
Funnel Plot.

Figure 2 shows the funnel plot of the 53 effect sizes, with standard error (SE) of the *g* on the vertical axis and the *g* values on the horizontal axis. Each black circle represents an individual effect size, plotted according to its estimated magnitude (*g*) and corresponding standard error. The plot indicates that the majority of effect sizes are evenly and symmetrically distributed around the mean, suggesting a low risk of publication bias and supporting the robustness of the findings.

Rosenthal’s Fail-Safe N indicated that 6790 missing or unpublished studies with null results would be required to cancel the overall effect size to non-significance. This number far exceeds 5 × *k* + 10 (in this case, *k* = 53, the result would be 275), further suggesting a low risk of publication bias and supporting the robustness of the findings.

Moreover, the Trim-and-Fill method yielded R_0_^+^ = 1 (<2) and L_0_^+^ = 0.44 (<3), indicating that the impact of publication bias was minimal and could be considered negligible.

### 4.3. Heterogeneity Test Results and Overall Effect

To examine the significance of the within-study variance (Level 2) and the between-study variance (Level 3), we conducted two fit tests comparing the fit of the three-level model (which included both within study and between-study variances) to the fit of two two-level models (one including only within-study variance, and the other including only between-study variance), respectively. Results are shown in Table 2. The proportion of total variance attributable to sampling variance (Level 1) was 11.47%, within-study variance (Level 2) accounted for 41.40%, and between-study variance (Level 3) for 47.13%. One-tailed log-likelihood ratio tests indicated that both Level 2 variance (*p* < 0.001) and Level 3 variance (*p* < 0.05) were significant, supporting the presence of substantial heterogeneity, confirming the appropriateness of employing a three-level random-effects model (Assink and Wibbelink 2016; Hunter and Schmidt 1990).

**Figure 3:**
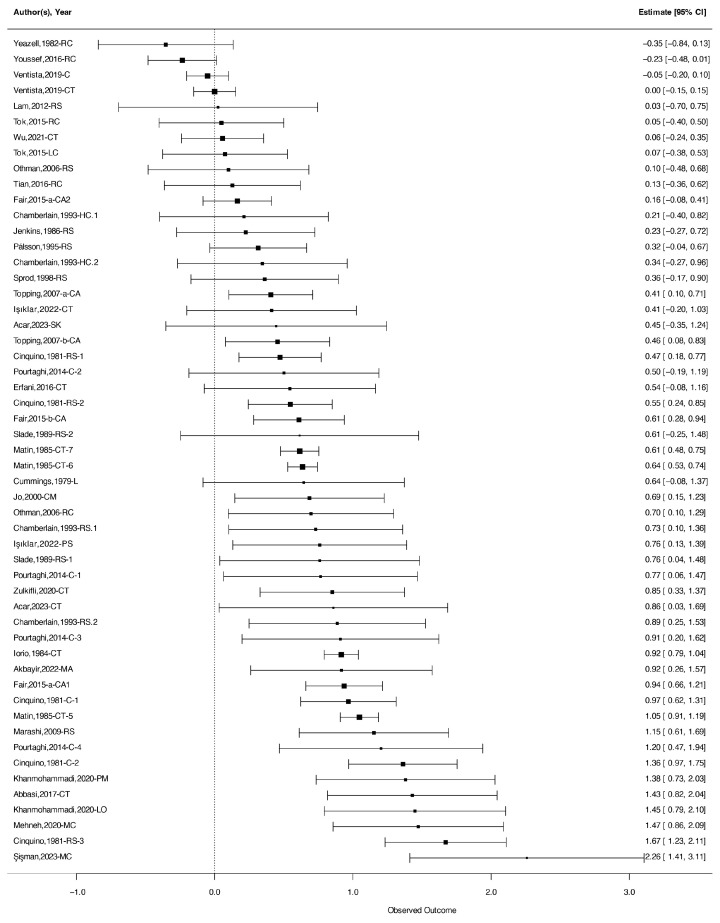
Forest plot based on a random-effect model displaying effect sizes with 95% confidence intervals. Notes: The studies included in the forest plot are as follows: Yeazell,1982-RC (Yeazell 1982); Youssef,2016-RC (Youssef et al. 2016); Ventista,2019-C (Ventista 2019); Ventista,2019-CT (Ventista 2019); Lam,2012-RS (Lam 2012); Tok,2015-RC (Tok and Mazl 2015); Othman,2006-RS (Othman and Hashim 2006); Tian,2016-RC (Tian and Liao 2016); Fair,2015-a-CA2 (Fair et al. 2015b); Chamberlain,1993-HC (Chamberlain 1993); Jenkins,1986-RS (Jenkins 1986); Pálsson,1995-RS (Pálsson 1995); Chamberlain,1993-HC (Chamberlain 1993); Sprod,1998-RS (Sprod 1998); Topping,2007-a-CA (Topping and Trickey 2007a); Işıklar,2022-CT (Işıklar and Öztürk 2022); Acar,2023-SK (Acar and Arslan 2023); Topping,2007-b-CA (Topping and Trickey 2007b); Cinquino,1981-RS-1 (Cinquino 1981); Pourtaghi,2014-C-2 (Pourtaghi et al. 2014); Erfani,2016-CT (Erfani and Rezaei 2016); Cinquino,1981-RS-2 (Cinquino 1981); Fair,2015-b-CA (Fair et al. 2015a); Slade,1989-RS-2 (Slade 1989); Matin,1985-CT-7 (Martin and Weinstein 1985); Matin,1985-CT-6 (Martin and Weinstein 1985); Cummings,1979-L (Cummings 1979); Jo,2000-CM (Jo 2000); Othman,2006-RC (Othman and Hashim 2006); Chamberlain,1993-RS (Chamberlain 1993); Işıklar,2022-PS (Işıklar and Öztürk 2022); Slade,1989-RS-1 (Slade 1989); Pourtaghi,2014-C-1 (Pourtaghi et al. 2014); Zulkifli,2020-CT (Zulkifli and Hashim 2020); Acar,2023-CT (Acar and Arslan 2023); Chamberlain,1993-RS (Chamberlain 1993); Pourtaghi,2014-C-3 (Pourtaghi et al. 2014); Iorio,1984-CT (Iorio et al. 1984); Akbayir,2022-MA (Akbayir and Tedikçi 2022); Fair,2015-a-CA1 (Fair et al. 2015b); Cinquino,1981-C-1 (Cinquino 1981); Matin,1985-CT-5 (Martin and Weinstein 1985); Marashi,2009-RS (Marashi 2008); Pourtaghi,2014-C-4 (Pourtaghi et al. 2014); Cinquino,1981-C-2 (Cinquino 1981); Khanmohammadi,2020-PM (Khanmohammadi et al. 2020); Abbasi,2017-CT (Abbasi et al. 2017); Khanmohammadi,2020-LO (Khanmohammadi et al. 2020); Mehneh,2020-MC (Mehnehj et al. 2020); Cinquino,1981-RS-3 (Cinquino 1981); Şişman,2023-MC (Şişman et al. 2023).

Findings revealed a moderate-to-large overall effect size (*g* = 0.59, 95% CI = [0.43, 0.75]), and the forest plot (Figure 3) illustrates the individual and pooled effect sizes.

### 4.4. Moderator Analyses

Subgroup analyses were conducted to examine potential moderators, including cognitive ability type (creativity, cognitive abilities, critical thinking, reading comprehension, reasoning skills, and others), session length (≤50 min vs. >50 min), session frequency (once per week vs. twice per week), total duration (≤20 weeks, >20 weeks), grade level (≤Grade 5 vs. ≥Grade 6), cultural context (Western vs. non-Western), and research design (RCT, Non-RCT controlled trial, and One-group pretest–posttest). Meta-regression tested publication year and sample size. Results are shown in Table 3.

Moderator analyses indicated that cognitive ability type [*F* (5, 47) = 1.91, *p* = 0.11], session length [*F* (2, 50) = 1.57, *p* = 0.22], weekly frequency [*F* (3, 49) = 0.10, *p* = 0.96], total duration [*F* (2, 50) = 0.90, *p* = 0.41], grade level [*F* (2, 50) = 1.02, *p* = 0.37], research design [*F* (2, 50) = 1.44, *p* = 0.247], and publication year [*F* (1, 51) = 0.15, *p* = 0.704] were all non-significant moderators.

One moderator was marginally significant: cultural context [*F* (1, 51) = 2.96, *p* = 0.09]. Effect sizes were larger in non-Western contexts (*g* = 0.74, 95% CI [0.50, 0.98]) than in Western contexts (*g* = 0.46, 95% CI [0.23, 0.68]).

Sample size emerged as a significant moderator [*F* (1, 51) = 4.07, *p* = 0.049], indicating that the cognitive effects of P4C decreased slightly as sample size increased (*β* = −0.001, *p* = 0.049).

## 5. Discussion

### 5.1. Overall Effects of P4C on Children’s Cognitive Abilities

To our knowledge, this study represents the first application of a three-level meta-analytic approach to systematically evaluate the impact of P4C on children’s cognitive abilities. The results indicate a moderate-to-strong and significant positive effect (*g* = 0.59, 95% CI = [0.43, 0.75], *p* < 0.001), confirming *H1*. This finding aligns robustly with prior empirical evidence (e.g., Acar and Arslan 2023; Jo 2000; Pourtaghi et al. 2014; Zulkifli and Hashim 2020).

P4C’s significant cognitive benefits can be effectively explained by its two foundational principles—introducing philosophy to children and the pedagogy of the Community of Inquiry (COI), both pioneered by Lipman and Sharp.

Bringing philosophy to children does not mean transmitting philosophical knowledge. Rather, it entails respecting and cultivating children’s innate philosophical curiosity, leveraging the intrinsic consonance between philosophy and sound thinking (Lipman 2003), and through philosophically rich stimulus, children are encouraged to raise and persistently explore questions of genuine interest, thereby stimulating sustained cognitive engagement.

The COI framework is deeply rooted in a rich tapestry of theoretical traditions, including Socratic dialogue, John Dewey’s epistemology of inquiry and political philosophy, Charles Sanders Peirce’s theory of inquiry, Justus Buchler’s theory of judgment, and the social constructivist perspectives of George Herbert Mead and Lev Vygotsky (Lipman 2003; Yarmel and Gregory 2025). Within a COI, children are encouraged to express their viewpoints on issues of common concern in a climate of respect and attentive listening. Crucially, each perspective is subject to collective critical scrutiny and rational evaluation. This ongoing dialectic provokes a wealth of cognitive activities, including conceptual definition, reasoning, exemplification and counter-exemplification, analogy-making, hypothesis identification, inference, the exploration of alternative possibilities, and so on. Through such dynamic social interaction, participants not only co-construct more reasonable and meaningful judgments, but also enhance their individual cognitive capacities and inquiry skills through communal thinking and inquiry. The latter is realized through what Vygotsky (1978) termed “internalization”, whereby higher mental functions are transformed from socially mediated interactions (interpsychical) into individualized cognitive functions (intrapsychical) (Lipman 2003; Kennedy 2013; Liu 2020; Gregory 2021; Yarmel and Gregory 2025).

Notably, the effect size obtained in this study (*g* = 0.59) was slightly larger than Yan et al. (2018)’s earlier meta-analysis (*d* = 0.43, 95% CI = [0.33, 0.53], *p* < 0.001). This divergence may be attributable to methodological advancements in the present study, including broader search strategies, access to more comprehensive databases, and an extended time span. These enhancements likely resulted in the inclusion of a larger, more diverse, and potentially more representative sample of primary studies. Crucially, the persistence of substantial effect underscores P4C’s enduring effectiveness over time. Despite evolving educational landscapes and methodological variations in research designs, contemporary studies continue to affirm the cognitive benefits of P4C.

### 5.2. Moderating Factors Influencing the Cognitive Effects of P4C

#### 5.2.1. Moderating Effect of Cognitive Domains

We found that the type of cognitive domain assessed did not significantly moderate the overall effect, failing to support *H2*. However, notable variations emerged across domains: P4C had significant positive effects on creativity, critical thinking, reasoning, and general cognitive abilities, but its effect on reading comprehension was small and non-significant.

Findings highlight P4C’s robust and significant positive effect on creativity (*g* = 0.72), a dimension unexplored in prior meta-analyses. This finding confirms theoretical expectations (Lipman 2003; Fisher 2007) and empirical studies (Lam 2021; Camhy and Iberer 1988; Naderi et al. 2012; Ghaedi et al. 2015). P4C cultivates a safe and open environment that values curiosity, questioning, and diverse perspectives over fixed answers. Through prompts like “Who has a new idea?” and “Could it be…?”, and by supporting children to express their thoughts through multiple modes including language, imagery, and embodied forms, P4C enables children to “think outside the box” (Lam 2021), thereby fostering their ability to make connections, generate novel ideas, explore multiple possibilities, and propose alternative solutions, ultimately promoting the multidimensional development of creativity (Fisher 2008; Akan and Çüçen 2023; Stokell et al. 2017).

The effect of P4C on critical thinking was moderate to large and significant (*g* = 0.66), in line with theoretical claims (Lipman 2003; Fisher 2008) and empirical evidence (Erfani and Rezaei 2016; Işıklar and Öztürk 2022). P4C also exerted a moderate to large effect on reasoning skills (*g* = 0.56). This aligns with Lipman (2003)’s theoretical expectations and earlier empirical findings (Lipman 1976; Karras 1979; Gasparatou and Kampeza 2012). This effect size is notably lower than Yan et al. (2018)’s earlier estimate (*d* = 1.02), which drew from two studies, whereas the present analysis is based on 13 effect sizes yielding from 9 studies. Notably, our result closely matches García-Moriyón et al. (2005)’s meta-analysis spanning 1976–2002 (*d* = 0.58), suggesting the reasoning benefit of P4C is robust. In P4C, facilitators and peers engage children by asking for questions and reasons, requesting examples or counterexamples, probing sources of information, examining assumptions and implications, identifying fallacies, and appealing to standards. These cognitive moves help children develop and internalize reasoning and critical thinking skills such as questioning, accommodating dissent, and self-correction, while also nurturing the intellectual virtues essential to critical thinking, including curiosity, fairness, open-mindedness, respect for others, and rationality (Lipman 2003; Fisher 2008; Shorer and Quinn 2023).

P4C also yielded a moderate, significant improvement in general cognitive ability (*g* = 0.51), mirroring the results of a ten-year longitudinal study by Colom et al. (2014), which reported an effect size of 0.44 for overall cognitive ability.

Specifically, P4C had a small and not significant effect on reading comprehension. Two possible explanations may account for this finding. First, P4C does not directly target reading skills, and improvements in reasoning may not immediately transfer to reading comprehension. As Ventista (2019) has suggested, “long-term implementation of P4C may positively influence academic performance”. Further large-scale longitudinal studies are needed to establish its effects on academic achievement. Second, the sensitivity and appropriateness of the measurement tools used may also contribute to the variability in results. For instance, Othman and Hashim (2006), who employed TOEFL—a test emphasizing reasoning—reported a moderate-to-large effect size (*g* = 0.6), whereas Tok and Mazl (2015), who used a self-designed test focusing more on literal comprehension, found a much smaller effect (*g* = 0.05). Although causality remains to be confirmed, this difference suggests that the design of measurement tools may influence the evaluation of outcomes. Future research would therefore benefit from adopting validated, sensitive, appropriate instruments that align closely with the targeted cognitive constructs, enabling a more accurate estimation of P4C’s cognitive effects.

Our findings confirm the positive effects of P4C, through the implementation of a community of inquiry, on children’s reasoning abilities, critical thinking, and creativity. While existing research has shown significant positive impacts of P4C on metacognitive skills (Şişman et al. 2023; Mehnehj et al. 2020) and problem-solving (Işıklar and Öztürk 2022), the limited number of studies included in this analysis prevented subgroup analysis in these areas. Future research should comprehensively incorporate more relevant analyses to accumulate sufficient studies for subgroup analyses in these areas.

#### 5.2.2. Moderating Effect of Dosage Variables

Among dosage-related variables, none showed statistically significant moderating effects, providing no support for *H3.1*, *H3.2*, or *H3.3*.

Nevertheless, descriptive patterns suggest that session length may still matter: sessions lasting ≤ 50 min yielded a moderate effect (*g* = 0.36, *p* < 0.05), whereas sessions extending beyond 50 min showed much larger effects (*g* = 0.65, *p* < 0.001). A typical P4C session typically includes text reading, question generation, and agenda-setting before discussion. Limited session duration may not provide sufficient time for participation, deeper thinking, and thorough dialogue, thereby reducing the effectiveness. While longer sessions appear more beneficial, practical constraints such as curriculum scheduling and student fatigue must be considered.

Subgroup analyses indicate no significant difference by frequency. Although intervention duration was not a statistically significant moderator, the pattern of effect sizes across subgroups is noteworthy. Shorter-term interventions (≤20 weeks) produced slightly higher effects (*g* = 0.61) than longer-term interventions (*g* = 0.49). One possible explanation is the Hawthorne effect—where the introduction of a novel and enthusiastically implemented program may temporarily enhance student engagement and performance, generating short-term gains that are difficult to sustain (Topping and Trickey 2015). These early effects, while encouraging, may not fully reflect the enduring impact of the intervention. Thus, even relatively brief P4C programs can produce meaningful benefits, but sustained implementation may help consolidate and stabilize these effects.

Due to missing data in the dosage variables, the generalizability of our findings may be limited, and care should be taken when generalizing the results.

#### 5.2.3. Moderating Effect of Grade Level

Findings of this study show that P4C exerts moderate-to-large positive effects on children’s cognitive abilities across all pre-college educational stages, with no significant moderating role of grade level (*p* = 0.37), thereby not providing support for *H4*. This aligns with Yan et al. (2018)’s finding, who also reported no evidence of grade-level moderation. It challenges a major objection to P4C—namely, the claim by Piaget (1933) and his followers that young children lack the higher-order thinking skills required for philosophical inquiry (Kitchener 1990; White 1992). Contrary to this assumption, our findings demonstrate that young children can engage in philosophical thinking and achieve significant cognitive gains through P4C. Our findings are congruent with contemporary developmental theories that view young children as capable learners and “philosophical babies”, offering evidence from a different perspective that even at an early age, children can engage in reasoning, reflection, and metacognitive activities. Moreover, the results demonstrate that young children can engage in philosophical thinking and achieve significant cognitive gains through P4C.

The consistent cognitive benefits of P4C across grade levels can be attributed to its pedagogy. Rather than transmitting simplified philosophical content, P4C creates an intellectual safe environment in which children are encouraged to explore philosophical questions arising from their lived experiences (Lipman et al. 1980; Matthews 1980), using everyday language rather than technical jargon. Such inquiry is accessible to learners of all ages, as it draws on their prior knowledge and experience. With teacher facilitation, students engage in cognitive moves such as evaluating reasons, offering examples, questioning assumptions, and forming reasoned judgments (Gregory 2008; Kennedy 2004). These dialogues promote development within each learner’s zone of proximal development, enabling even young children can engage in P4C and derive cognitive benefits.

#### 5.2.4. Moderating Effect of Cultural Context

Cultural context exhibited a marginally significant moderating effect (*p* = 0.09), with interventions implemented in non-Western contexts (*g* = 0.74) demonstrating larger effects than those in Western contexts (*g* = 0.46), supporting *H5*.

Although P4C originated in western philosophy, the findings highlight its cultural adaptability. As an educational program grounded in the community of inquiry, P4C maintains a clear theoretical core while allowing flexible adaptation. Including using culturally relevant stimulus—such as Confucian texts and idioms in China (Cao and Huang 2024; Lin and Huang 2025), or fairy tales in Denmark (Jespersen 2017); and context-sensitive pedagogical modifications, such as Jackson’s p4cHI model in Hawai’i, which aligns with local Aloha values (Jackson 2001, 2012). These localized practices enable P4C to resonate more deeply with context and demonstrate its compatibility with diverse educational settings. While implementation challenges exist—such as teacher-centered traditions and exam-driven systems in China (Ku 2008; Wu 2021; Lam 2019), “P4C has been proven to both necessitate and induce broader educational reforms (e.g., toward student empowerment, inquiry-based pedagogy)” (Gregory 2013), this suggests that P4C not only adapts to but also gradually transforms educational environments, supporting its applicability and long-term value across diverse cultures. For instance, Lam (2019) and Wu (2021) observed shifts toward more dialogical and child-centered practices, with positive outcomes captured through measurement and observation.

Consistent with Yan et al. (2018), the present study also found larger effects in non-Western than in Western contexts. We speculate that this difference may be partly driven by sample size effects. In this meta-analysis, non-Western studies (n = 17) involved an average of only 53 participants, whereas western studies (n = 16) averaged 229. Small-scale projects often benefit from more favorable implementation condition, such as greater researcher involvement, closer monitoring of fidelity, while large-scale studies are more susceptible to heterogeneity in delivery, including variation in teacher expertise, dosage, and classroom conditions (Yan et al. 2018). Baseline pedagogical differences may also amplify P4C’s impact. In many non-Western contexts, where education is shaped by collectivist values and higher power distance, instruction often emphasizes teacher authority, structured curriculum, and exam preparation, with limited space for critical thinking and autonomy (Lam 2012; Wu 2021; Imani et al. 2016; Kole 2025), the P4C pedagogy—child-centered, inquiry-driven, and encouraging critical reflection and student participation—may offer a more novel and engaging learning experience, potentially yielding greater gains in these contexts than in Western ones (Imani et al. 2016; Kiany and Movahedian 2010). Taken together, small-sample effects and baseline differences help explain this pattern, though the marginal significance (*p* = 0.09) calls for further larger cross-cultural evidence.

#### 5.2.5. Moderating Effect of Research Design

Findings of this study indicate that the type of research design did not significantly moderate the cognitive effects of P4C, which did not support *H6*. However, there was a gradient pattern: RCTs yielded the smallest effect size (*g* = 0.36), one-group pretest–posttest designs produced the largest (*g* = 0.88), and Non-RCTs fell in between (*g* = 0.57). This gradient aligns with expectations based on internal validity: RCTs minimize bias through random allocation, whereas one-group designs lack control conditions and are more susceptible to confounding factors, potentially inflating effect sizes. This finding somewhat supports García-Moriyón et al. (2005)’s conclusion that more rigorous designs tend to yield smaller, more conservative estimates. Given the relatively small number of RCTs included in this analysis (*k* = 4, *#es* = 5), the statistical power to detect moderating effects remains limited. Future research should prioritize well-controlled trials to better clarify the role of research design in shaping the estimated cognitive effects of P4C.

#### 5.2.6. Moderating Effect of Publication Year

Publication year did not emerge as a significant moderator (*β* = 0.002, *p* > 0.05) in this meta-regression model. This finding does not support *H7* and offers insight into the evolving landscape of P4C research. It may indicate a degree of temporal robustness in the cognitive benefits of P4C, suggesting that its effectiveness has persisted across decades.

#### 5.2.7. Moderating Effect of Sample Size

Meta-regression results revealed a significant negative relationship between sample size and effect size (*β* = −0.001, *p* < 0.05), indicating that smaller studies tended to report larger effects, supporting *H8*. This finding is consistent with the well-documented phenomenon of the small-study effects (Luo et al. 2020). In the context of educational interventions, large-scale studies often involve more implementation heterogeneity, including variation in teacher expertise, dosage, classroom conditions, and delivery consistency. For instance, Fair et al. (2015b) noted considerable discrepancies in session counts between grade levels in a large-scale P4C study, potentially contributing to uneven outcomes. These results highlight the importance of interpreting P4C effect sizes with attention to study scale, and the need for future research to incorporate detailed implementation monitoring to improve interpretability and robustness.

## 6. Conclusions, Limitation and Implications

### 6.1. Conclusions

This three-level meta-analysis of 33 studies (53 effect sizes) confirmed that P4C has a moderate-to-large, significant effect on children’s cognitive development, particularly in higher-order thinking skills such as creativity, reasoning, and critical thinking.

Subgroup and meta-regression analyses reveal that the cognitive effects of P4C are consistently positive across grade levels, research design, and publication years, indicating its cross-age, cross-design, and temporal stability. Dosage-related variables (session length, frequency, and total duration) were not significant moderators of cognitive outcomes, although descriptively longer sessions yielded larger effects than shorter ones. However, cultural context demonstrated marginally significant moderating trends, suggesting potential contextual and methodological influences that warrant further investigation. Sample size was found to be a significant moderator: smaller studies tended to report larger effect sizes.

### 6.2. Limitations

Despite its broad coverage, this meta-analysis has several limitations that future research should address.

First, although the included studies span over five decades and come from multiple regions, the geographic representation of the included studies was uneven, with concentration in Anglophone and select Asian countries. The exclusion of non-English publications may have omitted culturally distinct practices, limiting global generalizability. Future studies should incorporate multilingual databases and broaden regional coverage.

Second, although cultural context was examined as a moderator, our Western vs. non-Western coding is a heuristic simplification that overlooks within-group diversity. Country-level analyses were not feasible because several countries were represented by very few studies, limiting the validity of such comparisons. Future research should employ country-specific analyses to better capture how cultural values shape P4C’s effectiveness.

Third, although variations in measurement tools are a likely source of heterogeneity, their scattered and inconsistent use across studies made it unfeasible to treat them as a moderator in this meta-analysis. Future meta-analyses should systematically examine the role of measurement tools to account for variability in effect sizes, while primary studies should adopt validated and appropriately sensitive instruments that more accurately capture the targeted cognitive constructs that P4C is designed to foster, thereby enabling more precise estimates of P4C’s effects.

Fourth, several studies involved small sample sizes, which may have reduced statistical power and external validity. Large-scale studies are needed to provide more robust evidence.

Additionally, some moderator categories (e.g., RCTs, creativity outcomes) had few effect sizes, limiting the reliability of subgroup comparisons; missing data on key variables (e.g., intervention dosage) across studies introduced uncertainty, biasing analyses and reducing effect size precision. Future studies should broaden the coverage of underrepresented domains, and ensure comprehensive reporting of relevant data to enhance validity.

Finally, although key moderators were tested, other influential factors—such as teacher training quality and instructional fidelity—could not be tested due to limited reporting in primary studies. Future research should explore these factors through more detailed moderator analyses and mixed-methods approaches to uncover how these elements shape P4C’s effectiveness.

### 6.3. Implications

The present three-level meta-analysis may contribute to the field in several ways. First, by synthesizing 33 studies conducted over more than five decades, it provides the most comprehensive quantitative evidence to date on the cognitive effects of P4C. Second, to the best of our knowledge, this is the first study in the P4C field to employ a three-level meta-analytic approach instead of a traditional meta-analysis, improving the credibility of the findings. Third, this study also investigates moderating factors that have not been systematically examined in previous reviews, such as session length, weekly frequency, and creativity-related outcomes, offering new insights into the conditions that shape the cognitive impact of P4C. Taken together, this study not only provides robust evidence for the effectiveness of P4C but also offers important implications for policy-making, educational practice, and future research.

For educational policymakers and curriculum developers, the findings provide compelling evidence to consider P4C as a viable option for integrating into contemporary education systems. Its significant and consistent cognitive benefits—across grade levels, cultural contexts, and historical periods—highlight its broad adaptability and enduring relevance. Given the rising demand for higher-order thinking in today’s complex world—marked by information overload, rapid advances in artificial intelligence, growing value pluralism, and rising societal uncertainty, P4C can be adopted as a stand-alone subject or embedded within existing subjects to foster key cognitive competences.

For P4C practitioners, the results underscore the importance of thoughtful program design. Longer sessions (over 50 min) were linked to stronger cognitive gains, suggesting that adequate time is essential for effective inquiry. While P4C works across diverse settings, culturally responsive implementation—such as using locally relevant materials and age-appropriate facilitation—can further enhance its impact.

For researchers, the findings underscore the need for more rigorous and transparent empirical studies on P4C. Future research should use larger, more diverse samples to improve statistical power and generalizability. To enhance interpretability and implementation relevance, researchers are encouraged to report key contextual variables—such as teacher qualifications, training quality, instructional fidelity, and classroom interaction patterns. Incorporating process measures and mixed-method approaches can help unpack how and under what conditions P4C is most effective.

By fostering children’s engagement in a community of philosophical inquiry, P4C has once again been shown to enhance key cognitive competences such as critical thinking, reasoning, creativity and general cognitive abilities. As UNESCO (2007) affirms, P4C helps prepare individuals to “shoulder responsibilities in the face of the great challenges of the contemporary world”. Therefore, in the face of information overload, rapid advances in artificial intelligence, growing value pluralism, and rising societal uncertainty, P4C offers valuable insights for educational policy and curriculum reform, contributing to the development of reflective, responsible, and future-ready citizens.

## Figures and Tables

**Table 2:** Results of Heterogeneity Test and Overall Effects.

Model	*k*	*#es*	*N*	*t*	Hedges’ *g*	95%CI	%Var. at Level 1	%Var. at Level 2	%Var. at Level 3
REM	33	53	4568	7.20 ***	0.59	[0.43, 0.75]	11.47	41.40	47.13

**Notes:** REM, Random Effect Model; *k*, number of studies; *#es*, number of effect sizes; *N*, sample size; *t*, *t* test value for the difference between mean effect size and 0; Hedges’ *g*, effect sizes; CI, confidence interval; % Var, percentage of variance that is distributed at one of the three levels of the meta-analytic model. Level 1, sample variance; Level 2, variance between effect sizes from the same study; Level 3, variance between studies; ***, *p* < 0.001. The same below.

**Table 3:** Results of Moderator Analyses.

			*k*	*#es*	*g*	95% CIs	*t*_0_	*β*_1_	95% CIs	*t*_1_	*F*(*df*_1_, *df*_2_)
Cognitive Domains	Creativity		3	7	0.73	[0.33, 1.13]	3.66 ***				F(5, 47) = 1.91 *p* = 0.11
	Cognitive Abilities		4	5	0.51	[0.10, 0.92]	2.52 *	−0.21	[−0.56, 0.41]	−0.31	
	Critical Thinking		9	11	0.66	[0.37, 0.95]	4.54 ***	−0.07	[−0.53, 0.38]	−0.32	
	Reading Comprehension		5	5	0.08	[−0.33, 0.50]	0.40	−0.65	[−1.23, −0.07]	−2.26 *	
	Reasoning Skills		9	13	0.56	[0.27, 0.85]	3.94 ***	−0.17	[−0.63, 0.29]	−0.75	
	Others		10	12	0.82	[0.52, 1.13]	5.40 ***	0.09	[−0.41, 0.60]	−0.37	
Dosages	Session Length	≤50 min	9	12	0.36	[0.05, 0.67]	2.30 *				F(2, 50) = 1.57
		>50 min	18	31	0.65	[0.45, 0.87]	5.97 ***	0.29	[−0.09, 0.67]	1.53 *	
		Not reported	6	10	0.74	[0.37, 1.10]	4.06 ***	0.38	[−0.10,0.86]	1.58	
	Weekly Frequency	1 time	14	21	0.58	[0.31, 0.84]	4.34 ***				F(3, 49) = 0.10
		2 times	8	11	0.53	[0.17, 0.89]	2.92 *	−0.05	[−0.50, 040]	−0.21	
		others	2	5	0.59	[−0.08, 1.27]	1.26	0.02	[−0.70, 9.74]	0.05	
		Not reported	9	16	0.66	[0.33, 0.99]	4.04 ***	0.08	[−0.34, 0.51]	0.39	
	Intervention Duration	≤10 weeks	18	28	0.63	[0.40, 0.86]	5.42 ***				F(2, 50) = 0.90
		>20 weeks	13	21	0.49	[0.25, 0.74]	4.12 ***	−0.14	[−0.46, 0.19]	−0.83	
		Not reported	3	4	0.89	[0.30, 1.47]	3.03 **	0.26	[−0.38, 0.89]	0.82	
Grade	≤Grade 5		13	19	0.49	[0.25, 0.74]	4.00 ***				F(2, 50) = 1.02
	≥Grade 6		20	29	0.62	[0.41, 0.83]	5.96 ***	0.13	[−0.19, 0.44]	0.80	
	Mixed		1	5	0.97	[0.31, 1.64]	2.93 **	0.48	[−0.23, 1.19]	1.35	
Culture Context	Western		17	28	0.46	[0.23, 0.68]	4.10 ***				F(1, 51) = 2.96 *p* = 0.09
	non-Western		16	25	0.74	[0.50, 0.98]	6.17 ***	0.28	[−0.05, 0.61]	1.72	
Type of Study Design	RCT		4	5	0.36	[−0.11, 0.82]	1.55				F(2, 50) = 1.44
	Non-RCT		26	39	0.57	[0.39, 0.76]	6.17 ***	0.22	[−0.28, 0.72]	0.88	
	One-group pre-post		3	9	0.88	[0.45, 1.32]	4.06 ***	0.53	[−0.11, 1.16]	1.67	
Year			33	53				0.002	[−0.01, 0.01]	0.38	F(1, 51) = 0.15
Sample Size			33	53				−0.001	−[0.002, −0.000]	−2.02 *	F(1, 51) = 4.07 *

Notes: *t*_0_, *t* test value for the difference between mean effect size and 0. *β*_1_, estimated regression coefficient; *t*_1_, *t* test value for the difference between mean effect size and reference category; *F*(*df*_1_, *df*_2_), the result of the omnibus test; *, *p* < 0.05; **, *p* < 0.01; ***, *p* < 0.001.

## Data Availability

Coded data and scripts are available in the publicly accessible Open Science Framework (OSF) repository: https://doi.org/10.17605/OSF.IO/EUAPX (accessed on 1 October 2025).
